# Metagenomes and metagenome-assembled genomes from a nutrient removal plant at Los Angeles County Sanitation Districts (LACSD) that transitioned from high to low dissolved oxygen

**DOI:** 10.1128/mra.01494-25

**Published:** 2026-02-23

**Authors:** Blaise M. Enuh, Kevin S. Myers, Phil Ackman, Thomas Weiland, Natalie Beach, Michelle Young, Timothy J. Donohue, Daniel R. Noguera

**Affiliations:** 1 Great Lakes Bioenergy Research Center, University of Wisconsin-Madison 5228, Madison, Wisconsin, USA; 2 Wisconsin Energy Institute, University of Wisconsin-Madison, Madison, Wisconsin, USA; 3 Los Angeles County Sanitation Districts, Pomona Water Reclamation Plant, Pomona, California, USA; 4 Carollo Engineers, Westminster, Colorado, USA; 5 Department of Bacteriology, University of Wisconsin-Madison 205263, Madison, Wisconsin, USA; 6 Department of Civil and Environmental Engineering, University of Wisconsin-Madison 5228, Madison, Wisconsin, USA; Montana State University, Bozeman, Montana, USA

**Keywords:** metagenomics, microbial communities, wastewater treatment, dissolved oxygen

## Abstract

Operating biological nutrient removal (BNR) wastewater treatment plants with low dissolved oxygen (DO) conditions can reduce energy costs. We report on five metagenomes and 492 metagenome-assembled genomes (MAGs) obtained from samples collected at the Pomona water reclamation plant before and after a DO reduction from 3.5 to 0.7 mg/L.

## ANNOUNCEMENT

Effective nitrification and phosphorus removal can be achieved in biological nutrient removal (BNR) wastewater treatment plants operated with low dissolved oxygen (DO) levels (1–3). Changing from normal high-DO to low-DO operation can cause microbial community shifts (1, 4, 5). To advance insight into these changes, samples were collected at the Pomona water reclamation plant, Los Angeles County Sanitation Districts (LACSD) before and after an 18-month period during which the operating DO level was reduced from 3.5 to 0.7 mg/L (6). The plant treats an average of 9 million gallons per day (mgd) in a modified Ludzack-Ettinger biological nutrient removal (BNR) configuration with one anoxic and two aeration zones and uses model predictive control (MPC) technology and optical luminescent DO sensors for aeration control (6). In total, five mixed liquor samples were collected and analyzed: three from the high-DO condition and two from the low-DO condition.

After centrifugation to concentrate the microbial cells, DNA was isolated with the DNeasy PowerSoil Kit using the manufacturer’s protocol (Qiagen, Germantown, MD) and quantified via a Qubit fluorometer (Fisher Scientific, Waltham, MA, USA), then stored at −20°C prior to sequencing. DNA purity was assessed using a NanoDrop One spectrophotometer (Fisher Scientific), and concentration was determined with the Qubit dsDNA high-sensitivity assay (Fisher Scientific).

**Fig 1:**
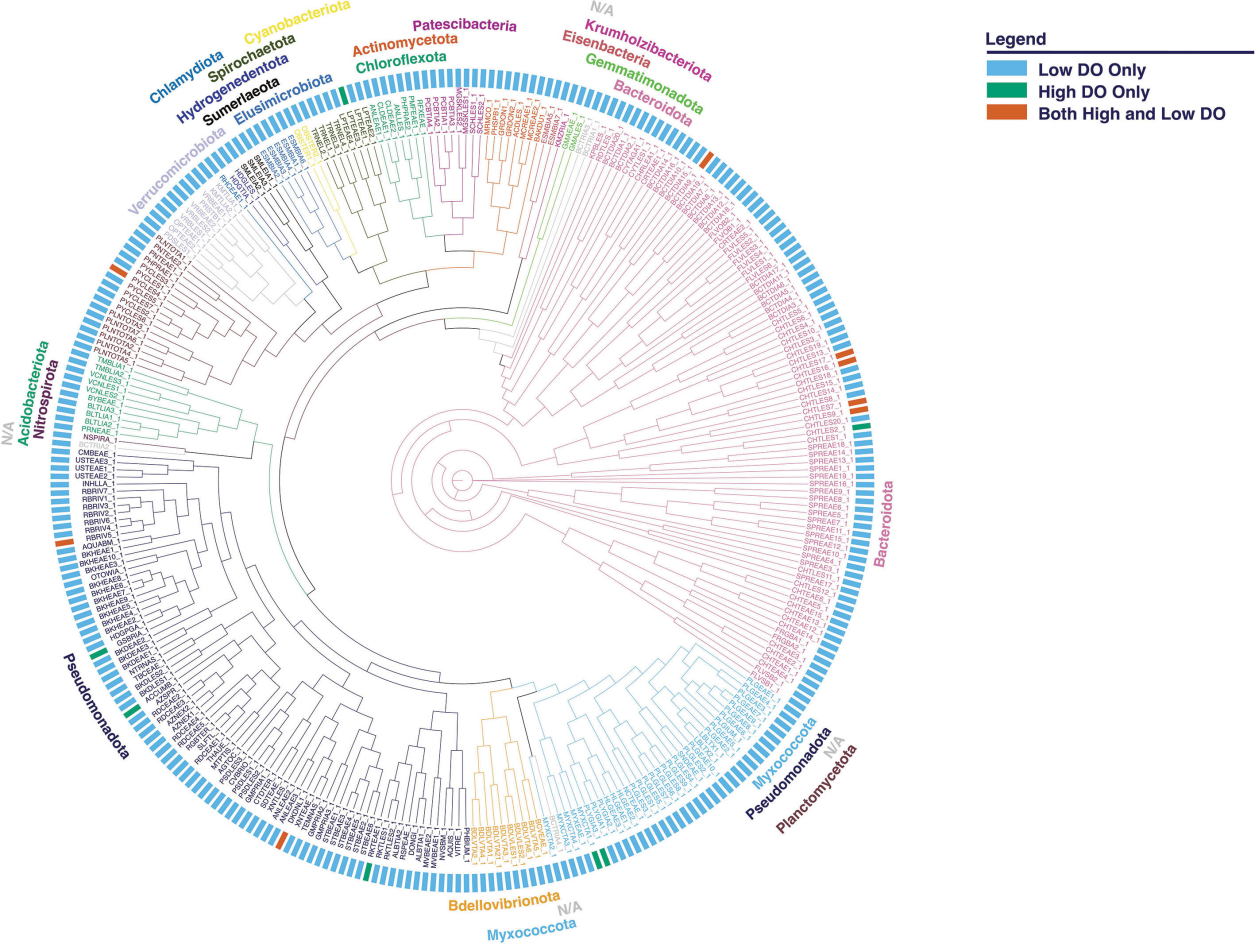
Phylogenetic map of 304 unique MAGs from the Pomona water reclamation plant. Each unique MAG represents a cluster of MAGs after dereplication. The outer ring is color-coded to indicate whether the cluster included MAGs from either low-DO or high-DO or both conditions. MAG classification is indicated with names matching the color of tree branches and with unclassified genomes labeled N/A.

HiFi library construction and sequencing were done at the University of Wisconsin-Madison Biotechnology Center (Madison, WI, USA). Libraries were generated following protocol PN 102-166-600 Version 04 (Pacific Biosciences, Menlo Park, CA, USA). Library integrity was evaluated on a FemtoPulse System (Agilent, Santa Clara, CA, USA), and quantification was done with the Qubit dsDNA high-sensitivity assay. Sequencing was done on a Sequel II platform with the Sequel Polymerase Binding Kit 2.2 following the standard protocol (Pacific Biosciences). Sequencing was implemented separately for the high-DO and low-DO samples. Unless otherwise stated, the following analyses were performed using default parameters. The circular consensus sequence (CCS) reads were assembled with metaFLYE (v2.9-b1768) (7) and metaMDBG (v0.3) (8), then polished with racon (v1.4.20) (9). The reads were mapped onto assemblies using minimap2 (v2.22-r1101) (10). Binning was done with metaBAT2 (v2:2.15) (11). Contaminated contigs were identified using both ProDeGe (v2.3) (12) and custom scripts for tetranucleotide frequency (run.GC.sh, Calculating_TF_Correlations.R; https://github.com/GLBRC/metagenome_analysis) and removed from the bins. All metagenome-assembled genomes (MAGs) were quality checked using CheckM (v1.2.2) (13), classified with GTDB-Tk (v2.1.0) database release 09-RS214 (14), and annotated with Bakta (v1.9.1) (15). RAxML-NG adaptive (v 1.2.1) (16) was used for maximum likelihood-based inference of the best phylogenetic tree (Fig. 1). The tree was visualized and further annotated using TreeViewer (v2.2.0) (17) and Inkscape (v1.2.2) (https://inkscape.org). We obtained a total of 492 MAGs, that is, 463 from the low-DO samples and 29 from the high-DO samples. Of these, 304 were deemed to be unique after dereplication with dRep (v0.6.1) (18) using a 95% identity cutoff. These data augment the knowledge base of microbial communities in BNR operated at low DO.

## Data Availability

Fastq files for the metagenomes have been deposited in the NCBI Sequence Read Archive (SRA) database under BioProject number PRJNA1333415. The MAGs, their characteristics, and annotations can be accessed on Figshare (10.6084/m9.figshare.30412474 [19]). Custom scripts used in the analyses can be found at https://github.com/GLBRC/metagenome_analysis.

## References

[1] Fitzgerald CM, Camejo P, Oshlag JZ, Noguera DR. 2015. Ammonia-oxidizing microbial communities in reactors with efficient nitrification at low-dissolved oxygen. Water Res 70:38–51. doi:10.1016/j.watres.2014.11.041 25506762 PMC4564296

[2] Keene NA, Reusser SR, Scarborough MJ, Grooms AL, Seib M, Santo Domingo J, Noguera DR. 2017. Pilot plant demonstration of stable and efficient high rate biological nutrient removal with low dissolved oxygen conditions. Water Res 121:72–85. doi:10.1016/j.watres.2017.05.029 28521237 PMC7388030

[3] Stewart RD, Bashar R, Amstadt C, Uribe-Santos GA, McMahon KD, Seib M, Noguera DR. 2022. Pilot-scale comparison of biological nutrient removal (BNR) using intermittent and continuous ammonia-based low dissolved oxygen aeration control systems. Water Sci Technol 85:578–590. doi:10.2166/wst.2021.630 35100140

[4] Park HD, Noguera DR. 2004. Evaluating the effect of dissolved oxygen on ammonia-oxidizing bacterial communities in activated sludge. Water Res 38:3275–3286. doi:10.1016/j.watres.2004.04.047 15276744

[5] Stewart RD, Myers KS, Amstadt C, Seib M, McMahon KD, Noguera DR. 2024. Refinement of the “Candidatus accumulibacter” genus based on metagenomic analysis of biological nutrient removal (BNR) pilot-scale plants operated with reduced aeration. mSystems 9:e0118823. doi:10.1128/msystems.01188-23 38415636 PMC10949500

[6] Young M, Beach N, Reifsnyder S, Ackman P, Weiland T, Dreher B, Bott C, Ekster A, Enuh BM, Noguera D, Kestel S, Khatiwada S, McCollough K, McIntosh L, Nagarkar M, Rosso D, Rauch-Williams T. 2025. Transforming aeration energy in water resource recovery facilities (WRRFs) through suboxic nitrogen removal (final report). 10.2172/2587564.

[7] Kolmogorov M, Bickhart DM, Behsaz B, Gurevich A, Rayko M, Shin SB, Kuhn K, Yuan J, Polevikov E, Smith TPL, Pevzner PA. 2020. metaFlye: scalable long-read metagenome assembly using repeat graphs. Nat Methods 17:1103–1110. doi:10.1038/s41592-020-00971-x 33020656 PMC10699202

[8] Benoit G, Raguideau S, James R, Phillippy AM, Chikhi R, Quince C. 2024. High-quality metagenome assembly from long accurate reads with metaMDBG. Nat Biotechnol 42:1378–1383. doi:10.1038/s41587-023-01983-6 38168989 PMC11392814

[9] Vaser R, Sović I, Nagarajan N, Šikić M. 2017. Fast and accurate de novo genome assembly from long uncorrected reads. Genome Res 27:737–746. doi:10.1101/gr.214270.116 28100585 PMC5411768

[10] Li H. 2018. Minimap2: pairwise alignment for nucleotide sequences. Bioinformatics 34:3094–3100. doi:10.1093/bioinformatics/bty191 29750242 PMC6137996

[11] Kang DD, Li F, Kirton E, Thomas A, Egan R, An H, Wang Z. 2019. MetaBAT 2: an adaptive binning algorithm for robust and efficient genome reconstruction from metagenome assemblies. PeerJ 7:e7359. doi:10.7717/peerj.7359 31388474 PMC6662567

[12] Tennessen K, Andersen E, Clingenpeel S, Rinke C, Lundberg DS, Han J, Dangl JL, Ivanova NN, Woyke T, Kyrpides N, Pati A. 2016. ProDeGe: a computational protocol for fully automated decontamination of genomes. ISME J 10:269–272. doi:10.1038/ismej.2015.100 26057843 PMC4681846

[13] Parks DH, Imelfort M, Skennerton CT, Hugenholtz P, Tyson GW. 2015. CheckM: assessing the quality of microbial genomes recovered from isolates, single cells, and metagenomes. Genome Res 25:1043–1055. doi:10.1101/gr.186072.114 25977477 PMC4484387

[14] Chaumeil PA, Mussig AJ, Hugenholtz P, Parks DH. 2019. GTDB-Tk: a toolkit to classify genomes with the Genome Taxonomy Database. Bioinformatics 36:1925–1927. doi:10.1093/bioinformatics/btz848 31730192 PMC7703759

[15] Schwengers O, Jelonek L, Dieckmann MA, Beyvers S, Blom J, Goesmann A. 2021. Bakta: rapid and standardized annotation of bacterial genomes via alignment-free sequence identification. Microb Genom 7:13. doi:10.1099/mgen.0.000685 PMC8743544 34739369

[16] Kozlov AM, Darriba D, Flouri T, Morel B, Stamatakis A. 2019. RAxML-NG: a fast, scalable and user-friendly tool for maximum likelihood phylogenetic inference. Bioinformatics 35:4453–4455. doi:10.1093/bioinformatics/btz305 31070718 PMC6821337

[17] Bianchini G, Sánchez-Baracaldo P. 2024. TreeViewer: flexible, modular software to visualise and manipulate phylogenetic trees. Ecol Evol 14:e10873. doi:10.1002/ece3.10873 38314311 PMC10834882

[18] Olm MR, Brown CT, Brooks B, Banfield JF. 2017. dRep: a tool for fast and accurate genomic comparisons that enables improved genome recovery from metagenomes through de-replication. ISME J 11:2864–2868. doi:10.1038/ismej.2017.126 28742071 PMC5702732

[19] Enuh B, Myers KS, Noguera DR. 2025. Metagenomes and metagenome-assembled genomes from microbial communities in the Los Angeles County Sanitation District (LACSD) biological nutrient removal pilot plants operated with high and low dissolved oxygen condition. Dataset. 10.6084/m9.figshare.30412474. PMC12981096 41728963

